# The Dietary Inflammatory Index Is Not Associated With Gut Permeability or Biomarkers of Systemic Inflammation in HIV Immunologic Non-responders

**DOI:** 10.3389/fnut.2021.736816

**Published:** 2021-11-22

**Authors:** Fat Malazogu, Rodney K. Rousseau, Nitin Shivappa, Sanja Huibner, Sharon L. Walmsley, Colin M. Kovacs, Erika Benko, Robert J. Reinhard, Ron Rosenes, James R. Hebert, Rupert Kaul

**Affiliations:** ^1^Departments of Medicine and Immunology, University of Toronto, Toronto, ON, Canada; ^2^Cancer Prevention and Control Program, University of South Carolina, Columbia, SC, United States; ^3^Department of Epidemiology and Biostatistics, Arnold School of Public Health, University of South Carolina, Columbia, SC, United States; ^4^Department of Nutrition, Connecting Health Innovations Limited Liability Corporation (LLC), Columbia, SC, United States; ^5^Department of Medicine, University Health Network, Toronto, ON, Canada; ^6^Maple Leaf Medical Clinic, Toronto, ON, Canada; ^7^Public/Global Health Consultant, Toronto, ON, Canada; ^8^Independent Researcher, Toronto, ON, Canada

**Keywords:** HIV—human immunodeficiency virus, Dietary Inflammatory Index, inflammatory biomarkers, immune non-responder, bacterial translocation

## Abstract

Immunologic non-responders (INRs) are a subset of individuals living with HIV who have suboptimal blood CD4+ T cell recovery despite effective antiretroviral therapy (ART). They are at an increased risk of serious non-AIDS co-morbidities and death, and demonstrate enhanced systemic immune activation. In other populations diet has been correlated with markers of systemic inflammation through the Diet Inflammatory Index (DII), but this association has not been studied in persons living with HIV (PLWH). Blood was collected from 28 INR PLWH with a blood CD4+ T cell count <350/μL despite ≥2 years of effective ART. Participants completed a Canadian Diet History Questionnaire, and their responses were used to calculate the DII. Plasma inflammatory markers (IFNγ, TNF, IL-6, sVCAM, D-dimer, sCD14 and CRP) were assayed by ELISA, cellular immune activation (HLA-DR and CD38 on CD4+ and CD8+ T cells) was quantified using flow cytometry, and small bowel permeability assessed by calculation of the urine LacMan ratio after drinking a mix of lactulose and mannitol. Participants were a median age of 57 years, had been on effective ART for 15 years, and the median DII was −1.91 (range of −3.78 to +2.23). No correlation was observed between DII and plasma markers of inflammation, levels of T cell activation, gut permeability, or the biomarker of bacterial translocation sCD14. Self-reported alcohol intake, a potential confounder of the relationship between diet and inflammatory biomarkers, was also not associated with systemic inflammation or gut permeability. Our findings suggest that other mechanisms, rather than diet, are likely to be the major driver of systemic inflammation in INR individuals.

## Introduction

Effective viral suppression by antiretroviral therapy (ART) among people living with HIV dramatically increases life expectancy and limits disease progression to AIDS (1). Despite this, treated HIV infection is associated with chronic immune activation and inflammation, which are in turn associated with early mortality and serious non-AIDS co-morbidities, such as neurocognitive, cardiovascular, and renal diseases (2–5). In addition, blood CD4+ T cell numbers are not restored in a subset of people living with HIV who successfully achieve viral suppression on ART; this immunologic non-response (INR) phenotype is clinically defined in various ways, although most often by peripheral blood CD4+ T cell count <350 /μL despite prolonged viral suppression on ART (6–8). CD8+ T cell activation is enhanced in INR individuals compared to clinical responders (CRs) who achieve a higher blood CD4+ T cell count during ART (8), and while the INR phenotype is incompletely understood this chronic immune activation may be why these individuals are at a higher risk of serious non-AIDS co-morbidities and mortality (9–11).

Gut epithelial barrier function is compromised in HIV-infected individuals (12), and the resulting increased gut-blood microbial translocation may be an important driver of host innate immune activation and inflammation (13, 14). Gut neutrophil infiltration and mucosal cell apoptosis remain elevated in ART-treated individuals living with HIV, more so in INR individuals (15), and this is thought to be associated with alterations in the gut microbiome (14). Therefore, restoring the gut microbiome and ameliorating gut epithelial damage is a potential target to reduce inflammation-mediated pathology amongst people living with HIV (16). In addition, alcohol use among adults infected with HIV may also be associated with decreased gut epithelial integrity and increased microbial translocation (17).

Diet is important for maintaining good health and also has important effects on the immune system and inflammation (18). “Western” diets, that have a high glycemic index and increased consumption of fat, red meat, and alcohol, have been linked to systemic inflammation (19–21). Meanwhile, diets that are lower in fat and enriched for fruits, vegetables, fiber and fish are associated with decreased inflammation (20, 22, 23). While mechanisms are insufficiently defined, diet composition reproducibly affects the gut microbiota and also influences microbial translocation (17, 24–26). The latter activates innate immune cells, including monocytes (27, 28), in part through the interaction of CD14 with bacterial lipopolysaccharide (LPS), LPS binding protein (LPB) and/or the MD-2/Toll-like receptor 4 (TLR4) signaling; this leads to the release of soluble CD14 (sCD14), which is measurable in blood plasma (27). Elevated plasma levels of sCD14 were linked with a Western dietary pattern and increased alcohol intake in the general population (26), and have also been associated with morbidity and mortality in people living with HIV (28).

While it is unclear whether diet contributes to systemic inflammation in the INR context, current HIV treatment guidelines recommend that, in the absence of other clinical strategies to improve CD4 counts or reduce inflammation, clinicians should promote diet-related interventions to modify risks for chronic disease (29). Therefore, a better understanding of the relationship between diet and inflammation in INR individuals will help to inform clinical approaches, and can be assessed via the Dietary Inflammatory Index (DII); if a relationship is apparent, then dietary interventions could also be tested as a clinical strategy to enhance health. The DII uses diet information to measure the inflammatory potential of diet (30–33), and we hypothesized that an elevated DII score (indicating a more pro-inflammatory diet) would be associated with increased plasma markers of inflammation and T cell activation in PLWH who were of the INR phenotype.

## Methods

### Participant Recruitment

Participants were recruited through the Maple Leaf Medical Clinic and the Immunodeficiency Clinic at the Toronto General Hospital (both in Toronto, Canada) as part of the PROOV IT 2 clinical trial of probiotic supplementation in INR individuals; the clinical trial protocol has been published elsewhere (16). The reported substudy of diet is based on assessment performed only at trial baseline, prior to administration of study product. Eligible participants were HIV-1 infected men over 18 years of age, who had taken ART and had an undetectable blood HIV-1 RNA (<50 copies/mL) for at least 2 years and had a most recent peripheral blood CD4 count <350/μl. Study exclusion criteria included colitis, liver fibrosis, clinical hepatitis, and/or portal hypertension. Regulatory approval of the study protocol was provided by Health Canada, and ethical approval by the research ethics boards of the University Health Network and the University of Toronto. All participants provided written, informed consent.

### Canadian Diet History Questionnaire

Dietary intake was assessed at study enrolment and completion, using the self-administered Canadian Diet History Questionnaire II (C-DHQ II) (34). The C-DHQ II was modified from the U.S. DHQ II, and is based on the Canadian Community Health Survey (CCHS), Cycle 2.2, Nutrition (2004) nutrient list. The C-DHQ II takes about 1 h to complete online and was administered either at home or on a computer at the clinic site. The C-DHQ II Dietary records were analyzed using the diet^*^calc 1.5.0 software (National Institutes of Health, https://epi.grants.cancer.gov/dhq2/dietcalc/). The nutrient database, comprised of 33 nutrients, was generated for each subject from 165 questions which query food, nutritional supplement use, and cooking of meat/vegetables over a 12-month period.

### Diet Inflammatory Index (DII^®^)

The DII is a validated method that uses diet composition data to calculate an output measure of the inflammatory effect of diet, as described previously (30). The inflammatory potential of the diet was calculated by computing the amounts of nutrients provided by the food frequency questionnaires. The mean intake of each food item was translated into statistical indices (*Z*-scores and centered percentiles), which were multiplied by their respective coefficients (overall food parameter-specific inflammatory effect scores) to calculate the DII score for each food parameter. Scores were summed to obtain the overall DII score for each participant. A positive DII score corresponds to a pro-inflammatory dietary habit and a negative DII score translates to an anti-inflammatory dietary habit.

Data collected through the C-DHQ II permitted 26 out of a possible 45 DII parameters from food and supplements to be used: alcohol, vitamin B12 and B6, B-carotene, caffeine, carbohydrate, cholesterol, energy, total fat, fiber, folic acid, iron, magnesium, monounsaturated fatty acids, niacin, protein, polyunsaturated fatty acids, riboflavin, saturated fat, selenium, thiamin, vitamin A, vitamin C, vitamin D, vitamin E, and zinc. Energy-adjusted DII scores were then obtained by converting food parameters from dietary intake to amount per 1,000 kcal; increasing positive scores indicate a pro-inflammatory diet, while decreasing scores indicate a more anti-inflammatory diet.

### Quantification of Plasma Biomarkers

Peripheral blood was collected into Acid Citrate Dextran (solution A) vacutainer tubes (BD Biosciences, Franklin Lakes, NJ USA) before being processed, with plasma stored directly at −80°C. Enzyme-linked immunosorbent assay (ELISA) kits were used to quantify plasma-soluble analytes in duplicate according to the manufacturers' instructions. Plasma concentrations of soluble vascular cell adhesion molecule (VCAM), C-reactive protein, and soluble CD14 were assayed by Quantikine^®^ Colorimetric Sandwich ELISA (R&D systems, Minneapolis, MN USA). Plasma concentration of D-dimer was assayed using IMUCLONE D-dimer ELISA (Sekisui Diagnostics, Stamford, CT USA). V-PLEX Human Proinflammatory Panel II (4-Plex; Meso Scale Discovery, Gaithersburg, Maryland, USA) was used to quantify interleukin (IL)-6, interferon (IFN)-γ and tumor necrosis factor (TNF), according to manufacturer's instructions. Samples were re-assayed if intra-assay coefficient of variability between duplicates was >20%CV and sample absorbance reading was outside the assay standard range.

### Flow Cytometry

Peripheral blood mononuclear cells (PBMCs) were isolated from fresh blood samples and used for the quantification of cellular activation by flow cytometry. PBMCs were stained with antibodies: BUV BUV395-anti-CD45 (BD Horizon), BV605-anti-CD3 (Biolegend), ECD-anti-CD4 (Beckman Coulter), APC-H7-anti-CD8 (BD Pharmingen), AF700-anti-CD38 (BD Pharmingen), BV785-anti-HLA-DR (Biolegend), as well as Aqua intracellular dye for dead cells (Invitrogen; L34957). Specimens were analyzed using a LSRFortessa X-20 cytometer (BD Biosciences), and inter-experiment consistency was calibrated using rainbow bead (Spherotech URFP-30-2). Proportional quantification of cellular activation subsets was done using FlowJo (Treestar, Woodburn, Oregon, USA).

### Intestinal Permeability

A urine-based lactulose:mannitol (LacMan) assay was used to assess intestinal permeability, as previously described (35). Prior to sleep participants consumed a drink containing a defined ratio of two saccharides, mannitol and lactulose, and they then collected all urine produced during the night and at the time of first void in the morning. Lactulose is not readily absorbed across the wall of the small intestine, while mannitol is readily absorbed, and so the LacMan ratio in urine was used as a proxy for small intestinal permeability. The concentration of each probe was measured in urine by high-pressure liquid chromatography.

### Statistical Analysis

Prism 8.3.1 software (Graphpad, San Diego, CA USA) was used for statistical analysis and figure production. Unadjusted Spearman analysis used to assess the relationships between DII score and immune markers, including the noted cellular and plasma-measured parameters.

## Results

### Participant Characteristics

Demographic characteristics for the 28 INR participants are displayed in Table 1. All participants were HIV-infected, had been treated with suppressive antiretroviral therapy for at least 2 years (median, 15 years) and had a plasma HIV-1 RNA viral load £40 copies/μl. Within the group the median (range) blood CD4+ T cell count was 249.5/μl (121–345/μl), and the median time since HIV diagnosis was 24 years (3–37 years; N=25). The median age or participants was 57 years (range 32, 76 years), and the DII score was −1.91 (−3.78, +2.23). Participants in the study were largely gay, bisexual and other men who have sex with men (MSM). Sixty-eight percent of participants were white, and their median body-mass index score (BMI) was 26.3 (18.8, 34.5) kg/m^2^.

**Table 1:** Participant clinical parameters and demographics (*n* = 28).

**Variable**	**Median**	**Range (min, max)**
Age (years)	57	32,76
Male, *n* (%)	28 (100)	
Race, *n* (%)		
Caucasian	19 (68)	
Black	2 (7.1)	
Asian	2 (7.1)	
Hispanic	2 (7.1)	
Other	3 (10.7)	
MSM, *n* (%)^a^	22 (92)	
*N* = 24		
DII	−1.91	−3.78, +2.23
Weight (kg)	82.9	55.7, 99.7
BMI (kg/m^2^)	26.3	18.8, 34.5
Nadir CD4 count (cells/ml)	42	3, 230
CD4 T cell count (cells/ml)	249.5	121, 345
CD8 T cell count (cells/ml)	571	101, 1,917
CD4/CD8 Ratio	0.435	0.11, 1.5
Time on ART (years)^a^		
*N* = 26	15	2,29
Years Since HIV diagnosis^a^		
*N* = 25	24	3,37
HIV RNA viral load	≤ 40	
Current smoker^a^, *N* = 23 (%)	2 (8.7)	
Previous smoker^a^, *N* = 23 (%)	10 (43.5)	
Number of ALCOHOLIC beverages (past 2 weeks)	2.5	0, 25

a *Incomplete sample set due to data availability; N shown*.

*MSM, men who report sex with men; DII, dietary inflammatory index; BMI, body mass index*.

### Diet Inflammatory Index and Soluble Biomarkers of Inflammation

**Figure 1:**
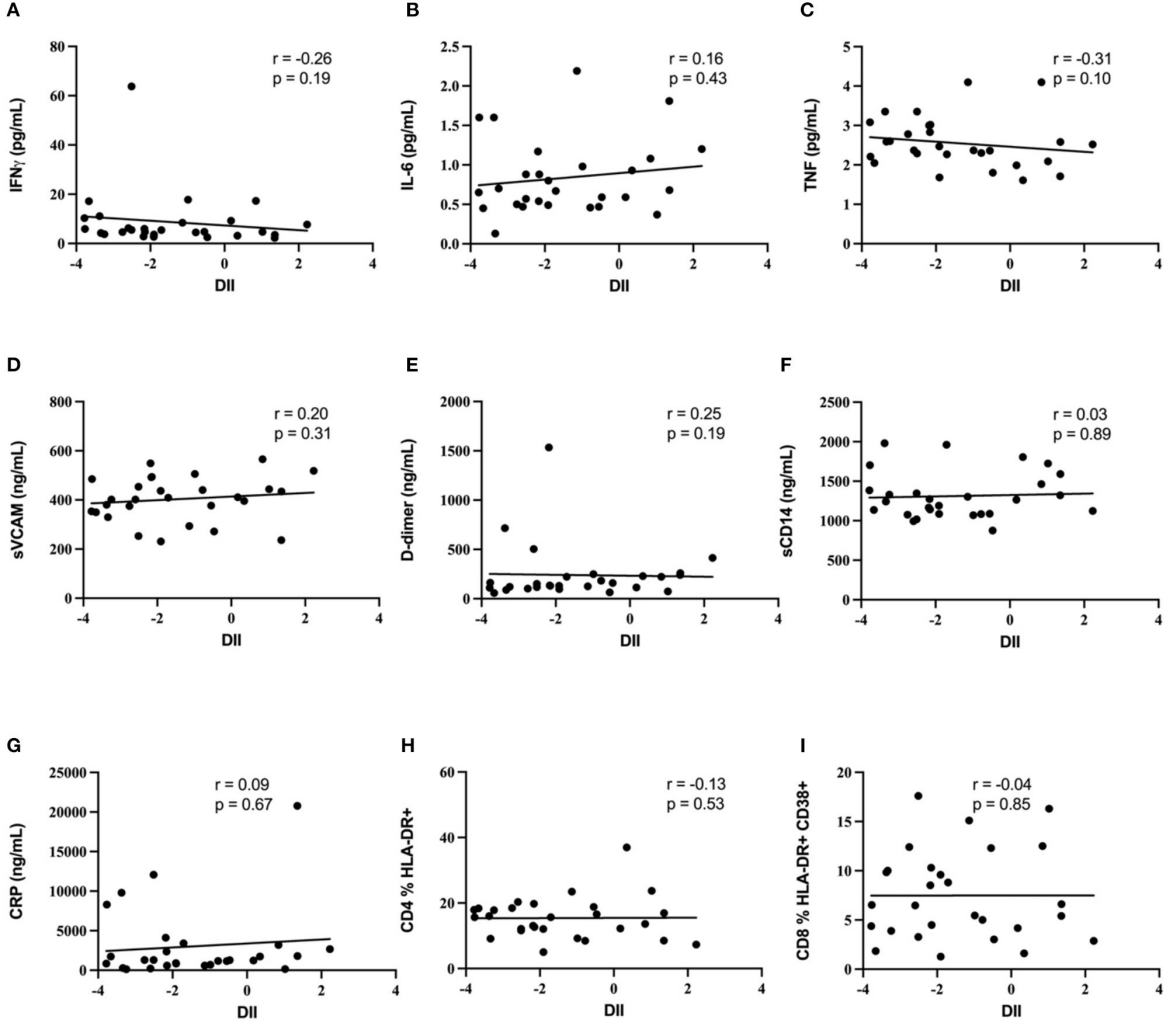
Diet inflammatory index and biomarkers of immune activation. Lack of correlation between diet inflammatory index and biomarkers of immune activation. No correlation was demonstrated between DII and **(A)** IFNg, **(B)** IL-6, **(C)** TNF, **(D)** sVCAM, **(E)** D-dimer, **(F)** sCD14, **(G)** CRP, **(H)** blood CD4 T cell activation, or **(I)** blood CD8 T cell activation.

The association of the diet inflammatory index with soluble plasma markers of inflammation (IFNγ, IL-6, TNF, sVCAM, CRP, and D-dimer) was then assessed, to test the hypothesis that an increased DII score would be positively associated with biomarkers of systemic inflammation in this INR cohort. Median values and ranges for these soluble immune parameters are shown in Table 2. Despite the relatively wide range of DII scores, no association was observed markers of inflammation IFNγ, IL-6, sVCAM, CRP or D-dimer (all p ≥ 0.15; Figure 1). In addition, the DII score tended to be negatively correlated with plasma levels of TNF (Spearman rho = −0.31, *p* = 0.10). Since vitamin A deficiency has been associated with immune activation in PLWH (36), we specifically assessed the correlation of estimated total vitamin A intake with biomarkers of systemic inflammation. Although there was a strong negative correlation between estimated vitamin A intake and DII score (Spearman rho = −0.66; *p* < 0.001), no correlation was seen with plasma levels of IFNγ, IL-6, sVCAM, CRP or D-dimer (all p ≥ 0.2).

**Table 2:** Summary of plasma inflammation associated biomarkers.

	**Median**	**SD**	**Range**
IFNg (pg/mL)	5.1	11.66	2.26–63.77
IL-6 (pg/mL)	0.67	0.47	0.13–2.19
TNF (pg/mL)	2.42	0.64	1.61–4.1
sVCAM (ng/mL)	405.46	91.19	230.79–565.84
D-dimer (ng/mL)	142.27	291.47	56.87–1534.98
sCD14 (ng/mL)	1254.77	293.68	875.76–1981.12
CRP (ng/mL)	1294.76	4477.85	127.19–20790.48
CD4 % HLA-DR+	15.69	6.37	5.02–36.96
CD8 %HLA-DR+ CD38+	6.5	4.56	1.28–17.6

*INFg, Interferon gamma; IL6, Interleukin 6; TNF, Tumor necrosis factor; sVCAM, soluble vascular adhesion molecule; D-dimer, is a fibrin degradation product; sCD14, soluble cluster of differentiation 14; CRP, C-reactive protein; CD4 % HLA-DR+, CD4 T cell activation; CD8 %HLA-DR+ CD38+, CD8 T cell activation*.

### Diet Inflammatory Index and T Cell Activation

**Figure 2:**
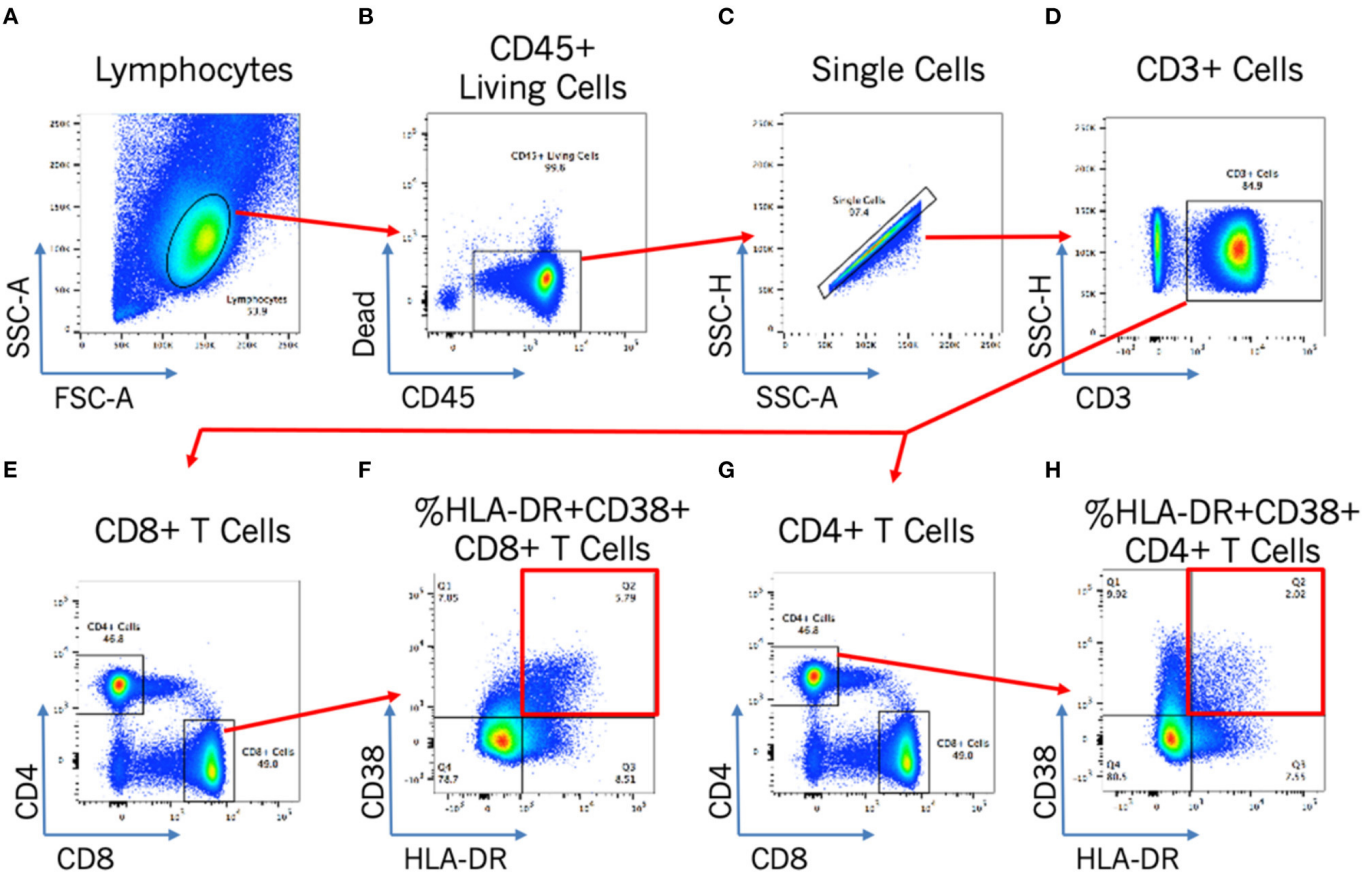
Flow cytometry gating strategy for T cell activation analysis. Flow cytometry gating strategy to identify by morphology and/or stain: lymphocytes **(A)**; CD45+ living cells **(B)**; single cells **(C)**; CD3+ cells **(D)**; and CD4+ or CD8+ activated cells [by HLA-DR+CD38+; **(E–H)**].

Blood T cell immune activation is increased in people living with HIV despite ART, and specifically within INR individuals, and so we next assessed the association of DII score with activation of both CD4+ T cells (CD4 %HLA-DR+) and CD8+ T cells (CD8 %HLA-DR+ CD38+). Representative plots in Figure 2 show the gating strategy that was used. There was no association between the DII score and blood T cell activation among INR participants (Figure 1), either with CD4+ T cell activation (CD4 %HLA-DR+; Spearman r = −0.13; *p* = 0.53) or CD8+ T cell activation (CD8 %HLA-DR+ CD38+; Spearman r = −0.4; *p* = 0.85). In addition, no association was seen between estimated vitamin A intake and either CD4+ or CD8+ T cell activation in the peripheral blood (both *p* > 0.4).

### Biomarkers of Gut Barrier Function and the Diet Inflammatory Index

Gut epithelial damage and bacterial translocation are increased in PLWH, even after long term ART, and are correlated with plasma levels of sCD14. We assessed gut epithelial damage using the overnight urine based LacMan assay and used sCD14 as a biomarker for bacterial translocation. The LacMan ratio and the plasma level of sCD14 were positively correlated with each other (*r* = 0.442; Spearman's *p* = 0.031), as would be expected if epithelial damage led to increased bacterial translocation. However, neither parameter was significantly associated with the diet inflammatory index (DII; *p* = 0.916 and *p* = 0.890, respectively).

### Self-Reported Alcohol Consumption and Gut Epithelial Integrity

**Figure 3:**
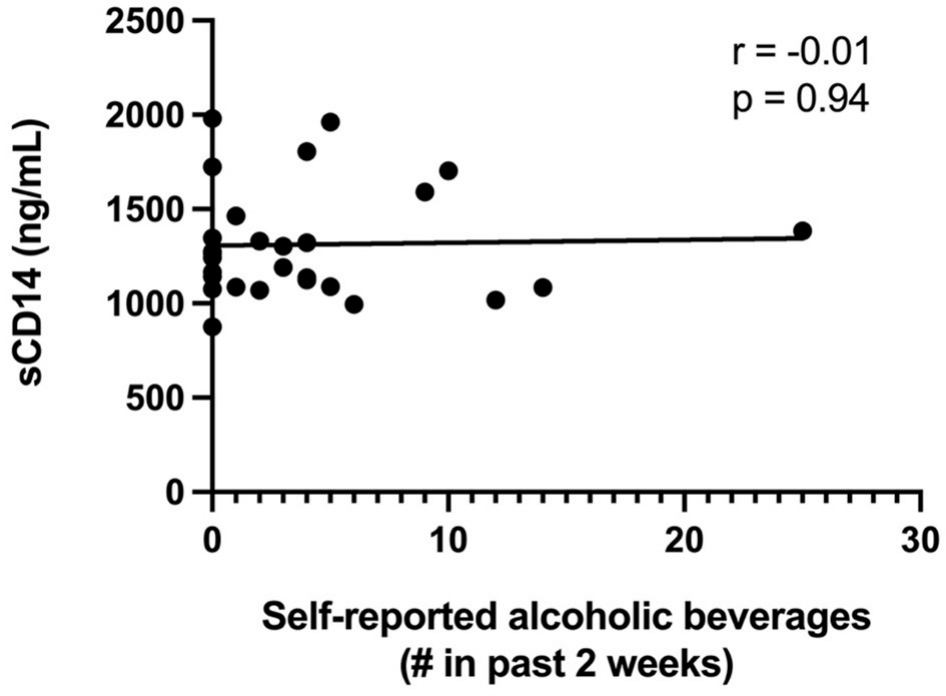
Plasma sCD14 concentrations and self-reported alcohol intake. Plasma sCD14 concentration does not correlate with self-reported alcoholic beverage consumption in the past 2 weeks. The self-reported number of alcoholic beverages (past 2 weeks) was correlated with plasma levels of the monocyte activation marker sCD14, a validated indicator of gut integrity and microbial translocation. An alcoholic beverage was defined as 5oz glass of wine, a bottle or can of beer, or 1oz of hard alcohol.

Since alcohol intake previously has been linked with decreased gut epithelial integrity in both humans and animal models (17, 37, 38), we analyzed the association between the self-reported intake of alcoholic beverages with both the LacMan ratio and the plasma level of sCD14, a validated indicator of gut integrity and microbial translocation. The median number (range) of alcoholic beverages reportedly consumed within the previous 2 weeks amongst study participants was 2.5 (0, 25). An alcoholic beverage was reported as the equivalent of a 5oz glass of wine, a bottle or can of beer, or 1oz of hard alcohol. We observed no association between alcohol consumption and either plasma sCD14 levels (*r* = −0.01, *p* = 0.94: Figure 3) or the LacMan ratio (−0.097, *p* = 0.653).

## Discussion

Despite effective antiretroviral therapy, elevated inflammation and immune activation persist in people living with HIV and are linked to serious non-AIDS co-morbidities and mortality (1–5). Immunologic non-response is commonly defined by a failure to restore normal CD4+ T cell counts in blood despite virus suppression on ART, and INR individuals are at a particularly high risk of serious non-AIDS co-morbidities and mortality (9–11). In addition, INR demonstrate elevated biomarkers of plasma and blood T cell activation when compared to general population, as well as to PLWH who have normalized their blood CD4+ T cell count on ART (8).

Diet has been linked to chronic diseases such as cardiovascular disease (18, 39, 40), and dietary interventions in HIV-uninfected participants consistently reduce immune activation and inflammation (39). Clinical guidelines suggest that INR individuals may benefit from following a “healthy diet,” with potential beneficial effects on CD4+ T cell counts and/or inflammation (29). However, although we found high levels of systemic inflammation and immune activation in this cross-sectional study of INR individuals from Toronto, Ontario, we were unable to demonstrate any association between inflammatory biomarkers or blood T cell activation and the DII score, a validated summary measure of dietary inflammatory potential (30–33). Vitamin A deficiency in particular has been linked to immune activation in PLWH (36), and although we found that estimated vitamin A intake was closely correlated with the DII score, this was again not correlated with increased T cell activation or elevated plasma inflammatory biomarkers.

These null findings do not mean that diet has no effects on host immunology, but they do suggest that the effect of diet on systemic inflammation may be relatively minor in the context of the very high levels of inflammation and immune activation that are found in people living with HIV, particularly within INR individuals. If this is the case, then dietary interventions targeting INR individuals would be unlikely to significantly improve inflammation-related health outcomes. However, larger-scale studies would be useful to assess whether these results can be generalized beyond the INR phenotype to HIV positive individuals with a more robust immune recovery on ART.

Alcohol use is common among adults infected with HIV (41), and has been associated with decreased gut epithelial integrity and increased microbial translocation in HIV-infected individuals, as evidenced by a link with increased plasma levels of LPS binding protein (LPB) and sCD14 (17). Therefore, we were concerned that alcohol intake, if associated with dietary differences, might confound our results, and so we explored the relationships between self-reported alcohol intake and immune endpoints. In this small study we found no relationship between self-reported alcohol intake and inflammatory biomarkers markers, including plasma sCD14. While this lack of association could indicate a lack of biological effect, it is also possible that it relates to the relatively narrow variation in alcoholic beverage consumption that was self-reported by our participants; this might either reflect a true low alcohol intake among individuals who were motivated to take part in our research, or systematic under-reporting of alcohol intake by participants due to desirability bias. Indeed, while our participants reported a median of 2.5 drinks per 2 weeks, much higher alcohol intake (22.1 ± 16.0 drinks per week) was reported in a prior study demonstrating a link between alcohol intake and plasma sCD14 levels among people living with HIV (42). Regardless, the lack of association in our cohort does mean that alcohol is unlikely to have acted as a confounder in our diet analysis.

There are limitations to our study that merit discussion. The study population consisted only of men, who were predominantly MSM, and so results may not apply to other populations, including women. In addition, the gut microbiome in MSM is enriched for pro-inflammatory bacterial species such as *Prevotella* (43, 44), potentially reducing our ability to demonstrate relationships between inflammation and other parameters such as diet. The relatively small sample size of our cohort limited our ability to perform a detailed multivariate analysis, and the inclusion only of INR individuals means that we could not assess potential correlations between diet and immune activation among PLWH who restore their CD4+ T cell count on ART, which would be an interesting area for future research. Within our study participant group, DII scores ranged from −3.78 to +2.23, a range that is somewhat narrower than prior population-based studies, such as the “The Cork and Kerry Diabetes and Heart Disease Study (phase II)” cohort (DII: −5.10 to +3.68) (33) and the “SUN study” cohort (DII: −5.14 to +3.97) (45). This may be explained by the narrow demographic breadth of the study population, which was constituted completely of men from Toronto-based HIV care sites. However, it seems likely that the observed DII range is representative of ART-treated PLWH in downtown Toronto, suggesting therefore that—in this context—diet is unlikely to be a major driver of the already-elevated inflammation and immune activation observed in INRs. Finally, as noted above, self-reported alcohol intake may underestimate true alcohol intake for several reasons, including desirability bias.

Our results should not be taken to mean that diet is unimportant to health. In the general population, diets meeting established recommendations, as assessed by diet quality measures, may delay the onset of co-morbidities prevalent in PLWH, including cardiovascular disease, diabetes, and neurodegenerative disease (46–48). Additionally, the prevalence of metabolic non-alcoholic fatty liver disease (NAFLD) and non-alcoholic steatohepatitis (NASH) is high among individuals living with HIV (49, 50). Diet modification, gradual weight loss, and vitamin E supplementation are currently recommended to improve parameters associated with NAFLD and NASH, although specific dietary recommendations are lacking (51). Our findings provide added nuance to our understanding of diet in the context of HIV, while not conflicting with current available guidance from the World Health Organization, which recommends that individuals consume a balanced healthy diet regardless of HIV status, and suggests that there is an urgent need to provide nutrition as a fundamental care package, given the devastating impact the HIV/AIDS epidemic has had on health, nutrition and food security (52). Nevertheless, in the absence of data suggesting that diet contributes to systemic inflammation among INR individuals, diet should not be a primary focus of strategies to improve immune health in this population.

## Data Availability Statement

The raw data supporting the conclusions of this article will be made available by the authors, without undue reservation.

## Ethics Statement

The studies involving human participants were reviewed and approved by University Health Network Research Ethics Board. The patients/participants provided their written informed consent to participate in this study.

## Author Contributions

FM, RKR, and RK conceptualized the study. FM performed statistical analysis and drafted initial manuscript. FM, RKR, and SH performed experiments and interpreted results. NS and JH performed diet inflammatory index calculations and participated in editing the manuscript. SW, CK, and EB enrolled and followed participants. RJR and RR were involved in participant enrollment and study acceptability. RKR and RK interpreted results and edited the draft. All authors reviewed and approved the final manuscript.

## Funding

Grant Support: Ontario HIV Treatment Network (OHTN, ROG 869), the Canadian Institutes of Health Research (CIHR; TMI 138656), and the Canadian HIV Trials Network (CTN) Pilot Funding Opportunity. RK was supported by a University of Toronto – OHTN HIV Research Chair. SW had a chair in HIV clinical management and aging from the OHTN.

## Conflict of Interest

JH owns controlling interest in Connecting Health Innovations LLC (CHI), a company that has licensed the right to his invention of the dietary inflammatory index (DII^®^) from the University of South Carolina in order to develop computer and smart phone applications for patient counseling and dietary intervention in clinical settings. NS is an employee of CHI. The remaining authors declare that the research was conducted in the absence of any commercial or financial relationships that could be construed as a potential conflict of interest.

## Publisher's Note

All claims expressed in this article are solely those of the authors and do not necessarily represent those of their affiliated organizations, or those of the publisher, the editors and the reviewers. Any product that may be evaluated in this article, or claim that may be made by its manufacturer, is not guaranteed or endorsed by the publisher.
